# The neural mechanisms underlying the aging-related enhancement of positive affects: electrophysiological evidences

**DOI:** 10.3389/fnagi.2015.00143

**Published:** 2015-08-06

**Authors:** Xianxin Meng, Jiemin Yang, AYan Cai, XinSheng Ding, Wenwen Liu, Hong Li, JiaJin Yuan

**Affiliations:** ^1^School of Education, Nanyang Normal CollegeNanyang, China; ^2^Faculty of Psychology, Southwest UniversityChongqing, China; ^3^Research Centre for Brain Function and Psychological Science, Shenzhen UniversityShenzhen, China

**Keywords:** aging, event-related potentials, positive affects, late positive potentials, attention

## Abstract

**Background:** Previous studies reported that old adults, relative to young adults, showed improvement of emotional stability and increased experiences of positive affects.

**Methods:** In order to better understand the neural underpinnings behind the aging-related enhancement of positive affects, it is necessary to investigate whether old and young adults differ in the threshold of eliciting positive or negative emotional reactions. However, no studies have examined emotional reaction differences between old and young adults by manipulating the intensity of emotional stimuli to date. To clarify this issue, the present study examined the impact of aging on the brain’s susceptibility to affective pictures of varying emotional intensities. We recorded event-related potentials (ERP) for highly negative (HN), mildly negative (MN) and neutral pictures in the negative experimental block; and for highly positive (HP), mildly positive (MP) and neutral pictures in the positive experimental block, when young and old adults were required to count the number of pictures, irrespective of the emotionality of the pictures.

**Results:** Event-related potentials results showed that LPP (late positive potentials) amplitudes were larger for HN and MN stimuli compared to neutral stimuli in young adults, but not in old adults. By contrast, old adults displayed larger LPP amplitudes for HP and MP relative to neutral stimuli, while these effects were absent for young adults. In addition, old adults reported more frequent perception of positive stimuli and less frequent perception of negative stimuli than young adults. The post-experiment stimulus assessment showed more positive ratings of Neutral and MP stimuli, and reduced arousal ratings of HN stimuli in old compared to young adults.

**Conclusion:** These results suggest that old adults are more resistant to the impact of negative stimuli, while they are equipped with enhanced attentional bias for positive stimuli. The implications of these results to the aging-related enhancement of positive affects were discussed.

## Introduction

Although human aging is associated with reductions of physical and cognitive abilities, many studies indicate that emotional stability and positive affects may enhance with normal aging (Mroczek and Kolarz, 1998; Ehrlich and Isaacowitz, 2002; Clark and Oswald, 2007). In an early study, Mroczek and Kolarz (1998) observed that old adults tend to report less negative experience and more positive experience than young adults. Consistent with this aging-related enhancement of emotional stability, a number of studies have demonstrated that relative to young adults, old adults showed preferential processing of positive information over negative information (Williams et al., 2006; Wood and Kisley, 2006; Kisley et al., 2007; Leclerc and Kensinger, 2008a; Feng et al., 2011), which is known as the aging-related positivity effect. Allard and Isaacowitz (2008) observed that old adults fixated more toward positive and neutral than negative pictures in both full and divided attention conditions. By recording eyeblink startle responses, Feng et al. (2011) reported that old adults showed potentiated responses when viewing positive pictures in comparison to negative pictures, whereas this was not the case for young adults. Socioemotional selectivity theory (SST) provides a framework for understanding the aging-related enhancement of positive affects. This theory states that motivation and goal preferences are influenced by time perspective (Carstensen et al., 1999). Young adults perceive their time remaining in life to be expansive and are more motivated to acquire knowledge whereas old adults perceive their time left in life as limited and would prioritize present-oriented goals of emotional meaning (Carstensen et al., 1999). This motivational shift leads to that old adults focus more attention on positive aspect of life.

A number of studies have investigated neural underpinnings of the aging-related enhancement of positive affects (Williams et al., 2006; Wood and Kisley, 2006; Kisley et al., 2007; Leclerc and Kensinger, 2008a). Using both event-related potential (ERP) and functional MRI methods, Williams et al. (2006) found that aging was associated with enhanced medial prefrontal activation for fearful faces and smaller activation for happy faces. The enhanced prefrontal control of negative and smaller control of positive information with aging has been considered as an important explanation for this phenomenon (Williams et al., 2006). In addition, several studies reported that the aging-related enhancement of positive affects is driven primarily by decreased neural responding to negative materials (Wood and Kisley, 2006; Kisley et al., 2007). For example, using an emotional categorization task and ERP technique, Kisley et al. (2007) observed age-related reductions in neural reactivity to negative pictures, but little age-related changes in neural reactivity to positive pictures. However, other studies demonstrated that this phenomenon is driven primarily by increased brain reactions to positive materials (Mather et al., 2004; Leclerc and Kensinger, 2008a). For example, by requiring subjects to identify the uses of emotional objects, Leclerc and Kensinger (2008a) observed greater activations of ventromedial prefrontal cortex for positive than for negative pictures in old adults, whereas young adults showed enhanced activations for negative than for positive pictures in this region.

Thus, there exist disagreements concerning the neural mechanisms behind the aging-related enhancement of positive affects. Though Williams et al. (2006) suggest that the aging- related shift in prefrontal control of negative and positive stimuli contributes to this phenomenon, this inference was based on a face perception task, instead of a direct emotional control task. Of particular importance, it needs to be elucidated whether this phenomenon is driven solely by decreased brain reactions to negative materials or by enhanced reactions to positive materials, or by both. It is worth noting that most of the prior studies which addressed neural mechanisms of this phenomenon required subjects to assess the emotionality of the stimuli explicitly (Mather et al., 2004; Williams et al., 2006; Wood and Kisley, 2006; Kisley et al., 2007; Leclerc and Kensinger, 2008a). It has been indicated that emotional effects are susceptible to the contamination of the explicit categorization of emotional stimuli, which is known as the “relevance-for-task effect” that is most pronounced in ERP experiment (Carretié et al., 1996, 2001). Thus, it is necessary to design a covert emotional task to control this confound. On the other hand, in order to obtain a clean emotional effect, it is necessary to set non-emotional, neutral stimuli as a baseline for positive or negative stimuli, and then to compute the emotion effect based on the emotional-neutral differences in dependent variables (Meng et al., 2009; Yuan et al., 2009), rather than simply comparing positive with negative stimuli.

Furthermore, in order to better understand the neural underpinnings behind the aging-related enhancement of positive affects, it is necessary to investigate whether old and young adults differ in the threshold of eliciting positive or negative emotional reactions. Specifically, because it is well established that aging is linked with better emotional stability and increased positive affects, it is possible that old adults are reactive to positive stimuli of low emotional intensity, which may not be the case for young adults. Similarly, given the robustness of this aging-related phenomenon, old adults are also likely to elicit emotional reactions to negative stimuli at a higher threshold than young adults. However, no studies have examined emotional reaction differences between old and young adults by manipulating the intensity of emotional stimuli to date. In fact, a great number of prior studies confirmed that the intensity of emotional stimuli is important (Leppǎnen et al., 2007; Yuan et al., 2007; Meng et al., 2009; Schaefer et al., 2009), and emotions of diverse intensities modulate cognitive activities differently (Yuan et al., 2008, 2012; Schaefer et al., 2009, 2011). Without manipulation of the emotional intensity of positive and negative stimuli, it is difficult to reveal the difference in the threshold of emotion elicitation across age groups.

In addition, a number of studies have indicated that the impact of aging on processing of emotional stimuli is clearly observed in controlled processing tasks which involve prefrontal cortex activity (Carstensen et al., 1999; Leclerc and Kensinger, 2008a); while this impact is not observed in tasks involving automatic processing (Hahn et al., 2006; Mather and Knight, 2006; Leclerc and Kensinger, 2008b; Mickley Steinmetz et al., 2010). For example, using eye-tracking method, Rosler et al. (2005) reported that when a negative–neutral picture pair was presented, young adults maintained attention longer toward negative pictures than old adults did, but young and old adults showed similar initial attentional orienting for the negative pictures. Using a spatial-cueing task, Brassen et al. (2011) reported that relative to young adults, old adults showed increased distractibility by happy faces in the high attention to faces condition. However, this age difference vanished when attention was low to faces (Brassen et al., 2011). Although these studies imply that the impact of aging on emotional processing entails the access of controlled processing resources (Leclerc and Kensinger, 2008a; Mickley Steinmetz et al., 2010; Sasse et al., 2014), how emotion-related aging effect varies with the information processing stages has yet to be directly investigated.

Based on the above considerations, the present study aimed to address the impact of aging on automatic and controlled processing of positive and negative stimuli of varying emotional intensity, by using ERP measures and a block-design covert-emotional task. We used ERP technique as it is helpful in depicting the timing features, specifically, the automatic and controlled processing of emotional stimuli and their modulation by aging. Previous studies used two ERP components, P1 and late positive potential (LPP), to reflect automatic and controlled processing, respectively (Wood and Kisley, 2006; Kisley et al., 2007; Olofsson et al., 2008; Langeslag and Van Strien, 2010). P1 component is an early component peaking about 100 ms post stimulus and has been accepted to reflect exogenous, automatic sensory processing (Scott et al., 2009; Dan and Raz, 2012). P1 amplitudes are thought to be sensitive to attention allocation (Luck et al., 1994; Smith et al., 2003; Brown et al., 2010), and be heightened for emotional stimuli compared to neutral stimuli (Scott et al., 2009; Dan and Raz, 2012). By contrast, LPP, which is also named late positive component (LPC) by some researchers (Ashley et al., 2004; Langeslag et al., 2007), starts around 400 ms and lasts for 700 ms. LPP reflects consciously controlled processing that involves continued voluntary attention toward emotional stimuli (Hajcak and Dennis, 2009; Langeslag and Van Strien, 2010). The LPP amplitudes increased with the enhancement of experienced emotion, and decreased with the reduction of experienced emotion (Flaisch et al., 2008; MacNamara et al., 2011). Based on previous studies (Carstensen et al., 1999; Hahn et al., 2006; Mather and Knight, 2006; Leclerc and Kensinger, 2008a,b; Mickley Steinmetz et al., 2010), we predict that the impact of aging on brain processing of emotional stimuli may occur at voluntary attention stage, instead of early automatic attention stage. Specifically, young and old adults may display similar emotional reactivity to emotional pictures in early P1 component whereas the two groups may display different emotional reactivity to pictures in LPP component.

In addition, considerable studies with young adults indicate that LPP elicited by negative pictures are largest over the parietal scalp (Cuthbert et al., 2000; Hajcak and Olvet, 2008; Yuan et al., 2014a,b). Based on these evidences, if old adults relative to young adults show decreased negative emotional reactivity, it is likely to observe decreased LPP amplitudes for negative pictures at parietal scalp, in old compared to young adults. On the other hand, functional MRI studies have indicated that the processing bias of old adults for positive stimuli is mainly manifested by the greater neural activity for positive relative to negative stimuli in prefrontal cortical regions (e.g., ventromedial or dorsolateral PFC; Leclerc and Kensinger, 2008a; Ritchey et al., 2011). However, the covert emotional task is associated with similar late potentials for positive and neutral stimuli in young adults (Yuan et al., 2007, 2009). Based on these evidences, we hypothesize to observe enhanced LPP amplitudes for positive compared to neutral pictures at prefrontal scalp, in old adults but not in young adults.

On the other hand, previous studies often used a random design, which presented positive, negative and neutral stimuli in a single block, with their order fully randomized (Wood and Kisley, 2006; Kisley et al., 2007). Though these studies observed clear emotion effects in electrophysiological measures, they observed no emotional effect in behavioral measures (Wood and Kisley, 2006; Kisley et al., 2007). A possible reason is that presenting positive to negative (or negative to positive) stimuli across trials may produce inter-trial emotion offset, which prevents the generation of a robust emotion induction effect in behavioral assessment. However, it is important to assess emotion impact from behavioral measures, not only for confirming whether a given type of emotional stimuli effectively elicit the target emotion, but also for verifying what neurophysiologic results truly reflect. For these considerations, the current study used a block design (Rowe et al., 2007; Yuan et al., 2012), with one block presenting highly positive (HP), mildly positive (MP) and neutral pictures while the other block presenting highly negative (HN), mildly negative (MN) and neutral pictures. A neutral baseline condition was used in either block, to isolate the emotional effect for each condition. To investigate the behavioral index of emotion impact and its relation with aging, subjects were asked to report the perceived frequency and the category of emotional images for each block, according to subjective impressions. We used this procedure, rather than direct mood rating, to avoid a potential floor effect in mood data because emotional stimuli were intermixed with neutral stimuli in either block. If old adults truly differ from young adults in susceptibility to positive or negative stimuli, the perceived frequency should be different across age groups. This method has been verified effective in reflecting group differences in susceptibility to emotional stimuli in our prior study (Yuan et al., 2009).

Lastly, in order to avoid cultural bias when International Affective Picture System (IAPS) was used directly in Chinese subjects (Huang and Luo, 2004), the pictures used to elicit emotional responses in the present study were selected from the native Chinese Affective Picture System (CAPS), which was established in a similar way to IAPS (Bai et al., 2005). According to the widely accepted dimensional theory of emotion, the affective significance of a stimulus is organized along the two primary dimensions: valence and arousal (Lang et al., 1997; Bradley et al., 2001). Intense emotional stimuli are normally accompanied by higher arousal in comparison with mildly emotional stimuli, irrespective of whether the stimuli are positive or negative (Bradley et al., 1990; Lang et al., 1997; Cuthbert et al., 2000; Keil et al., 2002; Kuppens et al., 2013). Thus, we predict that HP pictures would be rated more positive and more arousing than MP pictures; and HN pictures be rated more negative and more arousing than MN pictures.

## Materials and Methods

### Subjects

As paid volunteers, 17 young adults (age: 18–22; 8 females) and 17 old adults (age: 60–74; 9 females) in local community were randomly sampled for the experiment. The gender composition was not significantly different across the two samples [χ^2^ (1) = 0.118, *p* = 0.73]. All the subjects were right-handed and had no self-reported visual problems. As a final check of visual function, all the subjects read textual instructions on a computer screen at a distance of 2.5 m easily. In addition, both young and old adults were healthy, reporting no current symptoms and no history of anxious or depressive disorders. No subjects were taking medication that would affect the central nervous system. Each participant signed an informed consent form prior to the experiment. The experimental procedure was in accordance with the ethical principles of the 1964 Declaration of Helsinki (World Medical Organization [WHO], 1996). Prior to the experiment, the life satisfaction (LS) was assessed by the subjects answering “how is your life recently?” ranging from 1: very stressful; 3: ordinary to 5: very good. The scores of young adults were not significantly different from 3 [*t*(16) = 1.098, *p* = 0.289], while old adults’ rating was higher than 3 [*t*(16) = 4.243, *p* = 0.001]. The LS was higher for old (4.055) relative to young adults [3.294; *t*(32) = –2.088, *p* = 0.045).

### Stimulus Materials

**FIGURE 1:**
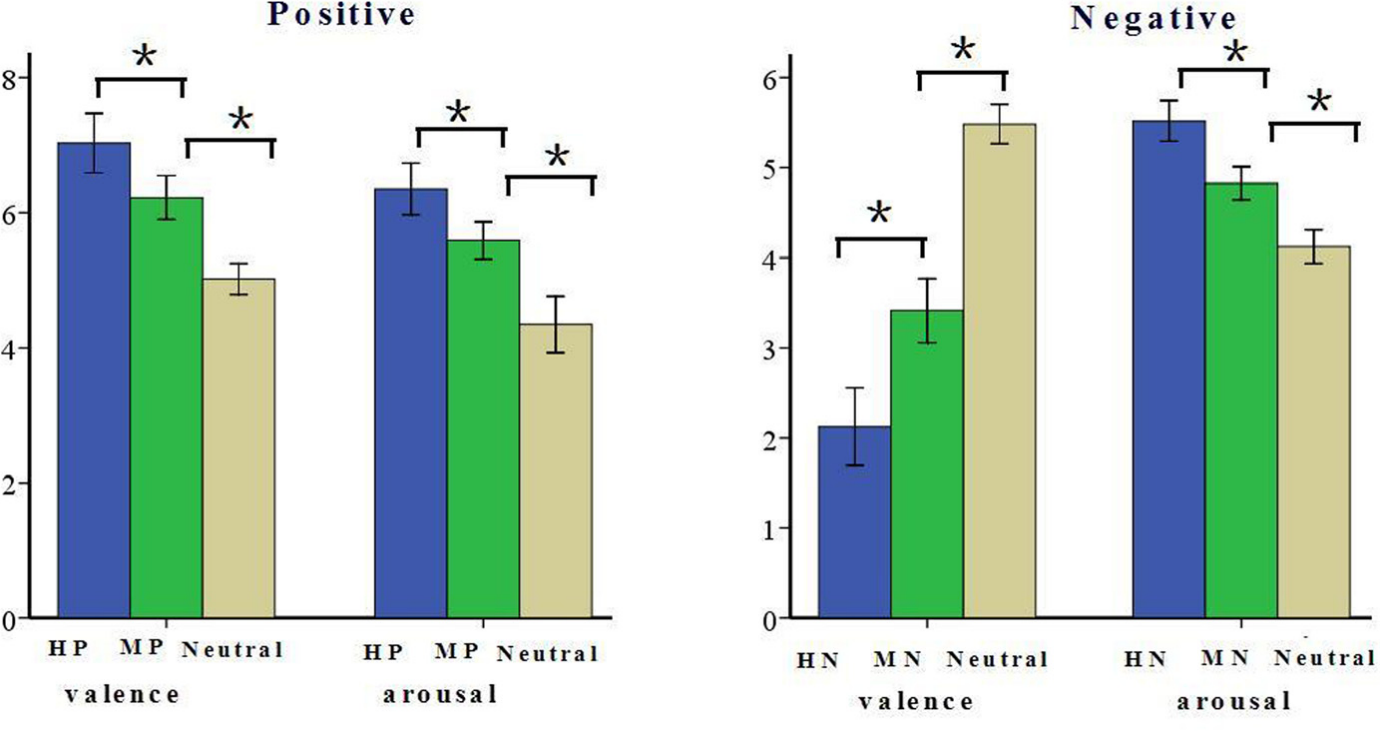
**Schematic illustration of the means and SD of valence and arousal assessments based on Normative Ratings (Bai et al., 2005).** ^∗^*p* < 0.05.

The present study included two experimental blocks. Each block consisted of 168 pictures (grouped into three conditions). In the positive block, 168 pictures were grouped as HP, MP, or neutral. Like many other studies using IAPS, the pictures used for this study covered a variety of contents (Cuthbert et al., 2000; Smith et al., 2003), such as highly pleasant, mildly pleasant, or neutral animals (e.g., puppies, pandas, or wolfs), natural scenes (e.g., landscapes, seashores, or mountains) and human activity (e.g., cheers, sports, conversation). In the negative block, 168 pictures were grouped as HN, MN, or neutral; such as HN, MN, or neutral animals (e.g., snakes, bugs, or eagles), natural scenes (e.g., fire disaster, flood, clouds) and human activity (e.g., homicide, violence, or sports). All the 336 pictures were selected from the CAPS. In the positive block, the three sets of pictures differed significantly from one another in both valence [*F*(2,165) = 286.14, *p* < 0.001] and arousal [*F*(2,165) = 290.54, *p* < 0.001]. HP pictures were rated more positive than were MP pictures [*F*(1,110) = 86.62, *p* < 0.001] which, in turn, were rated positive compared with the Neutral pictures [*F*(1,110) = 121.63, *p* < 0.001]. Also, HP pictures were rated more arousing relative to MP pictures [*F*(1,110) = 98.76, *p* < 0.001] which, again, were rated more arousing than were Neutral stimuli [*F*(1,110) = 145.49, *p* < 0.001]. In the negative block, the three sets of pictures differed significantly from one another in valence [*F*(2,165) = 1348.33, *p* < 0.001] and arousal [*F*(2,165) = 615.69, *p* < 0.001]. HN pictures were rated more negative than were MN pictures [*F*(1,110) = 293.65, *p* < 0.001] which, in turn, were rated negative compared with the Neutral pictures [*F*(1,110) = 1337.01, *p* < 0.001]. Also, HN pictures were rated more arousing relative to MN pictures [*F*(1,110) = 273.37, *p* < 0.001] which, again, were rated more arousing than were Neutral pictures [*F*(1,110) = 379.2, *p* < 0.001, see **Figure 1**]. All the pictures were identical in size and resolution (15 cm × 10 cm, 100 pixels per inch). In addition, the luminance level of the pictures was tested prior to experiment, and the luminance level and spatial frequency were matched across the three conditions in each block. The contrast of the monitor was set to a constant value across subjects.

### Behavioral Procedures

Subjects were seated in a quiet room at approximately 150 cm from a computer screen with the horizontal and vertical visual angles below 6°. Subjects were required to count the number of pictures. Each trial was initiated by a fixation cross for 1000 ms. The offset of the fixation was followed by the presentation of picture stimulus for 1000 ms. The inter-trial interval ranged randomly between 800 and 1000 ms. Each of the 336 pictures was just presented for once during the experiment. Between the two experimental blocks, 3 min of rest was used to prevent fatigue. The present study used E-Prime software (Psychology Software Tools, Pittsburgh, PA, USA) to control the presentation and timing of all stimuli. Each picture was displayed in color and occupied the entire screen of a 19-in. (48.26 cm) monitor at a 60-Hz refresh rate with a resolution of 1024 × 768 pixels of the screen. Each subject participated in both experimental blocks, with order of the blocks counterbalanced across subjects. In the rest period, subjects were asked to report the perceived frequency by percentage and the category of emotional images in negative and positive blocks, respectively, according to their subjective impressions. After the EEG recording, subjects were asked to rate the valence and arousal of the pictures using the Self-Assessment Manikin procedure (SAM; Lang et al., 1997). Using a self-report nine-point rating scale, subjects were required to rate the emotion valence (ranging from 1 = “very negative” to 9 = “very positive”) and arousal (ranging from 1 = “very calm” to 9 = “very excited”) they felt for each image by pressing corresponding number keys in the keyboard. The sequence of the two ratings was counterbalanced across subjects.

### ERP Recording and Analysis

The EEG was collected on 64 scalp sites using tin electrodes mounted in an elastic cap (Brain Products), with the references on the left and right mastoids (average mastoid reference, Luck, 2005) and a ground electrode on the medial frontal aspect. Vertical electrooculograms (EOGs) were recorded above and below the left eye. Horizontal EOG was recorded from the left versus right orbital rim. EEG and EOG activity was amplified at a bandpass of DC∼100 Hz and digitized with a sampling rate of 500 Hz. The EEG was filtered between 0.01 and 16 Hz. EEG recording did not start until all electrode impedances were kept below 5 kΩ. ERP averages were computed off-line; Trials with EOG artifacts (mean EOG voltage exceeding ±80 μV) and those contaminated with artifacts due to amplifier clipping, or peak-to-peak deflection exceeding ±80 μV were excluded from averaging.

EEG activity in each condition was averaged separately. ERP waveforms were time-locked to the onset of stimuli and the averaging epoch was 1200 ms, including a 200 ms pre-stimulus baseline. We selected the following nine electrode sites for statistical analysis of LPP amplitudes (400–1000 ms): FPz, FP1, FP2 (three prefrontal sites), Cz, C3, C4 (three central sites), Pz, P3, and P4 (three parietal sites). A repeated measures ANOVA of mean LPP amplitudes was conducted with the following repeated factors: emotion intensity (three levels: HN, MN, and neutral for negative block; HP, MP, and neutral for positive block), frontality (three levels: prefrontal, central, and parietal), and laterality (three levels: left, midline, and right) in negative and positive block, separately. Aging was used as a between-subjects factor. In order to explore the timing features of LPP modulation in old and young samples, the LPP waveform was quantified by mean amplitude measures in three time windows: 400–600 ms, 600–800 ms, 800–1000 ms, as recommended by prior studies (Hajcak and Nieuwenhuis, 2006; Foti and Hajcak, 2008). On the other hand, the mean amplitudes of occipital P1 (70–130 ms) were analyzed at O1, Oz, and O2 (three occipital sites), to explore the effects of aging on early visual processing. A repeated measures ANOVA was performed with emotion, block, electrode and aging as factors. The degrees of freedom of the *F*-ratio were corrected according to the Greenhouse–Geisser method in all these analyses. The *post hoc* pairwise comparisons were conducted using Bonferroni–Holm correction method if a significant main or interaction effect was detected.

## Results

### Cognitive Performances during the Counting Task

In the negative block, 15 old adults and 16 young adults accurately reported the number of pictures. In the positive block, 14 old adults and 15 young adults accurately reported the number of pictures. The proportion of subjects who accurately reported the number of pictures was not significantly different between the two samples in both negative [χ2 (1) = 0.366, *p* = 0.545] and positive [χ2 (1) = 0.234, *p* = 0.628] blocks. These results suggest that the two age groups did not differ in cognitive performances during the counting task.

### Emotion Assessment

**FIGURE 2:**
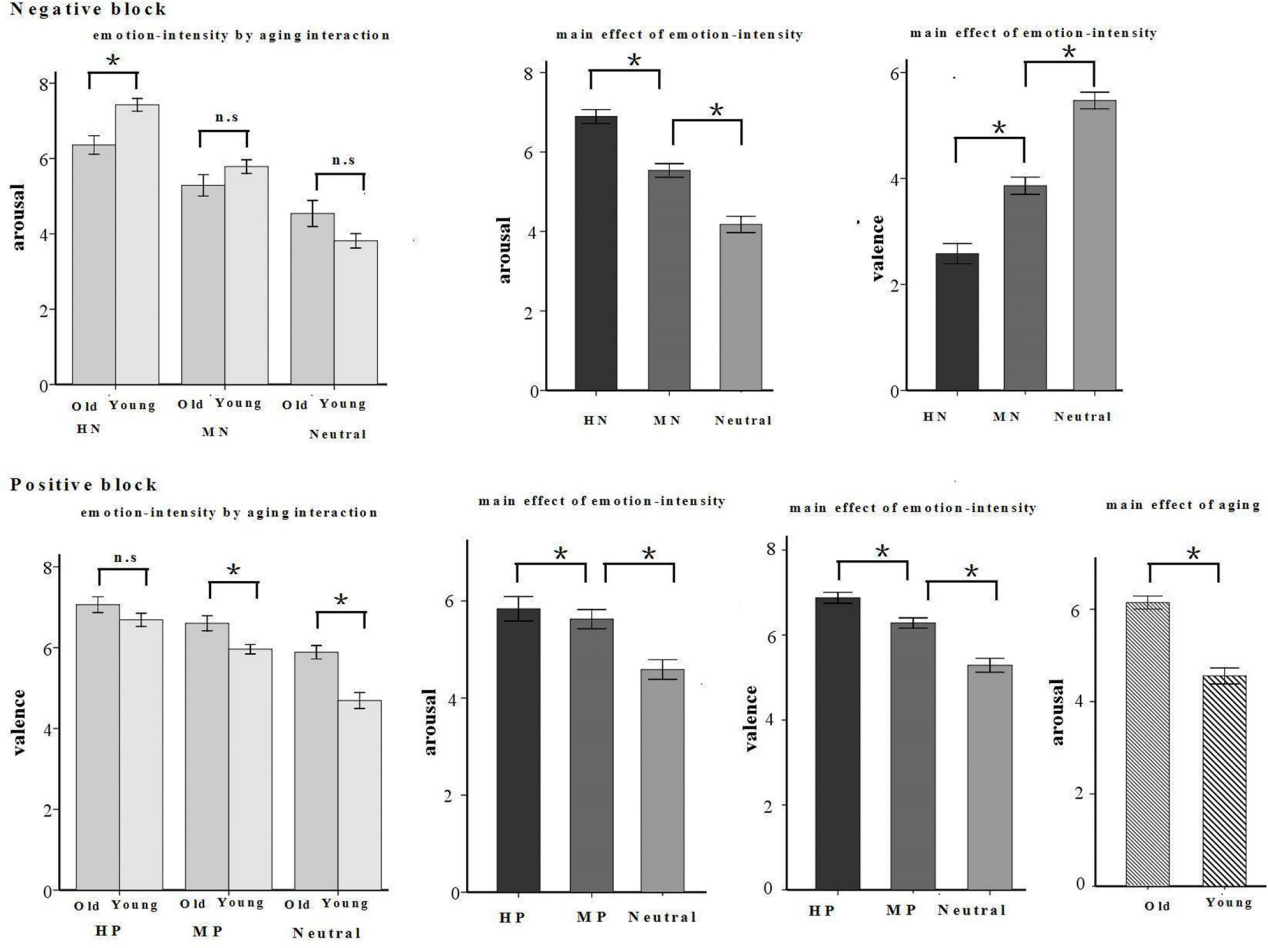
**Schematic illustration of means and SE of valence and arousal assessments given by young and old participants.** ^∗^*p* < 0.05.

A repeated measures ANOVA of arousal and valence ratings was conducted with the following factors: emotion intensity (three levels: HN, MN, and neutral for the negative block; HP, MP, and neutral for the positive block), and age (young, old). The results in the negative block showed a significant main effect of emotion intensity in valence rating [*F*(2,64) = 104.949, *p* < 0.001]. HN pictures were rated more negative than were MN pictures [*F*(1,32) = 91.824, *p* < 0.001] which, in turn, were rated negative compared with the Neutral pictures [*F*(1,32) = 79.229, *p* < 0.001]. Also, there was a significant main effect of emotion intensity in arousal rating [*F*(2,64) = 158.217, *p* < 0.001]. HN pictures were rated more arousing relative to MN pictures [*F*(1,32) = 217.687, *p* < 0.001] which, again, were rated more arousing than were Neutral stimuli [*F*(1,32) = 80.864, *p* < 0.001]. In addition, there was a significant interaction between emotion intensity and aging in arousal rating [*F*(2,64) = 14.087, *p* < 0.001]. The breakdown of the interaction shows that the arousal rating was higher in young than in old adults in HN [*F*(1,32) = 12.748, *p* < 0.01], but not in Neutral [*F*(1,32) = 2.161, *p* = 0.151] and MN [*F*(1,32) = 2.191, *p* = 0.149] pictures (see **Figure 2**).

The results in the positive block showed a significant main effect of emotion intensity [*F*(2,64) = 6.657, *p* < 0.01] and a significant interaction between emotion intensity and aging [*F*(2,64) = 98.026, *p* < 0.001] in valence rating. HP pictures were rated more positive than were MP pictures [*F*(1,32) = 67.25, *p* < 0.001] which, in turn, were rated pleasant compared with the Neutral pictures [*F*(1,32) = 74.407, *p* < 0.05]. The breakdown of the interaction shows that old adults rated MP [*F*(1,32) = 8.537, *p* < 0.01] and Neutral [*F*(1,32) = 21.215, *p* < 0.001] pictures, but not HP pictures [*F*(1,32) = 2.153, *p* = 0.152], as more positive than young adults. Also, there were significant main effects of emotion intensity [*F*(2,64) = 56.159, *p* < 0.001] and aging [*F*(1,32) = 26.689, *p* < 0.001] in arousal rating. HP pictures were rated more arousing relative to MP pictures [*F*(1,32) = 6.107, *p* < 0.05] which, again, were rated more arousing than were Neutral stimuli [*F*(1,32) = 67.314, *p* < 0.001]. Regardless of emotion intensity, all the pictures were rated more arousing by old adults than by young adults (see **Figure 2**).

### ERP Results

**FIGURE 3:**
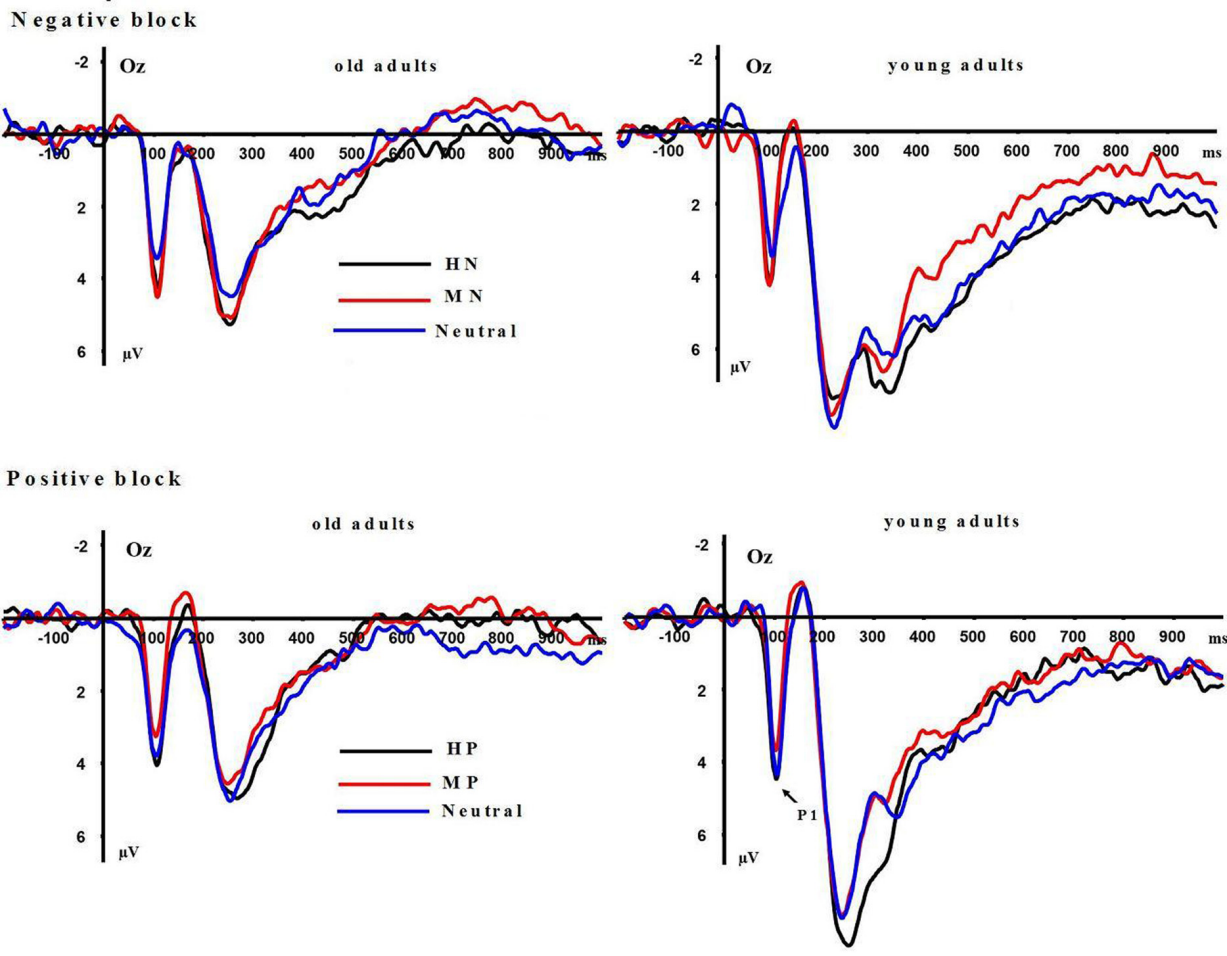
**Averaged event-related potentials (ERPs) at Oz site for highly emotional, mildly emotional, and neutral picture sets in young and old adults during positive and negative blocks**.

**P1:** A repeated measures ANOVA on P1 amplitudes showed significant main effects of emotion intensity in the negative block [*F*(2,64) = 10.999, *p* < 0.001] and in the positive block [*F*(2,64) = 8.536, *p* < 0.01]. In the negative block, both HN [*F*(1,32) = 9.25, *p* < 0.001] and MN [*F*(1,32) = 11.36, *p* < 0.001] stimuli elicited larger amplitudes than Neutral stimuli, while the amplitudes were similar during HN and MN conditions [*F*(1,32) = 0.362, *p* > 0.50], irrespective of age groups. In the positive block, HP stimuli elicited larger amplitudes than MP [*F*(1,32) = 17.137, *p* < 0.001] and Neutral stimuli [*F*(1,32) = 4.976, *p* < 0.05], while the amplitudes were similar during MP and Neutral conditions [*F*(1,32) = 2.652, *p* = 0.113], regardless of age groups (see **Figure 3**). Main effects of aging were not observed in the negative [*F*(1,32) = 0.001, *p* = 0.992] and positive [*F*(1,32) = 0.116, *p* = 0.735] blocks. No significant interaction effects between aging and emotion intensity were observed in the negative [*F*(2,64) = 0.104, *p* = 0.895] and positive [*F*(2,64) = 0.186, *p* = 0.819] blocks.

**FIGURE 4:**
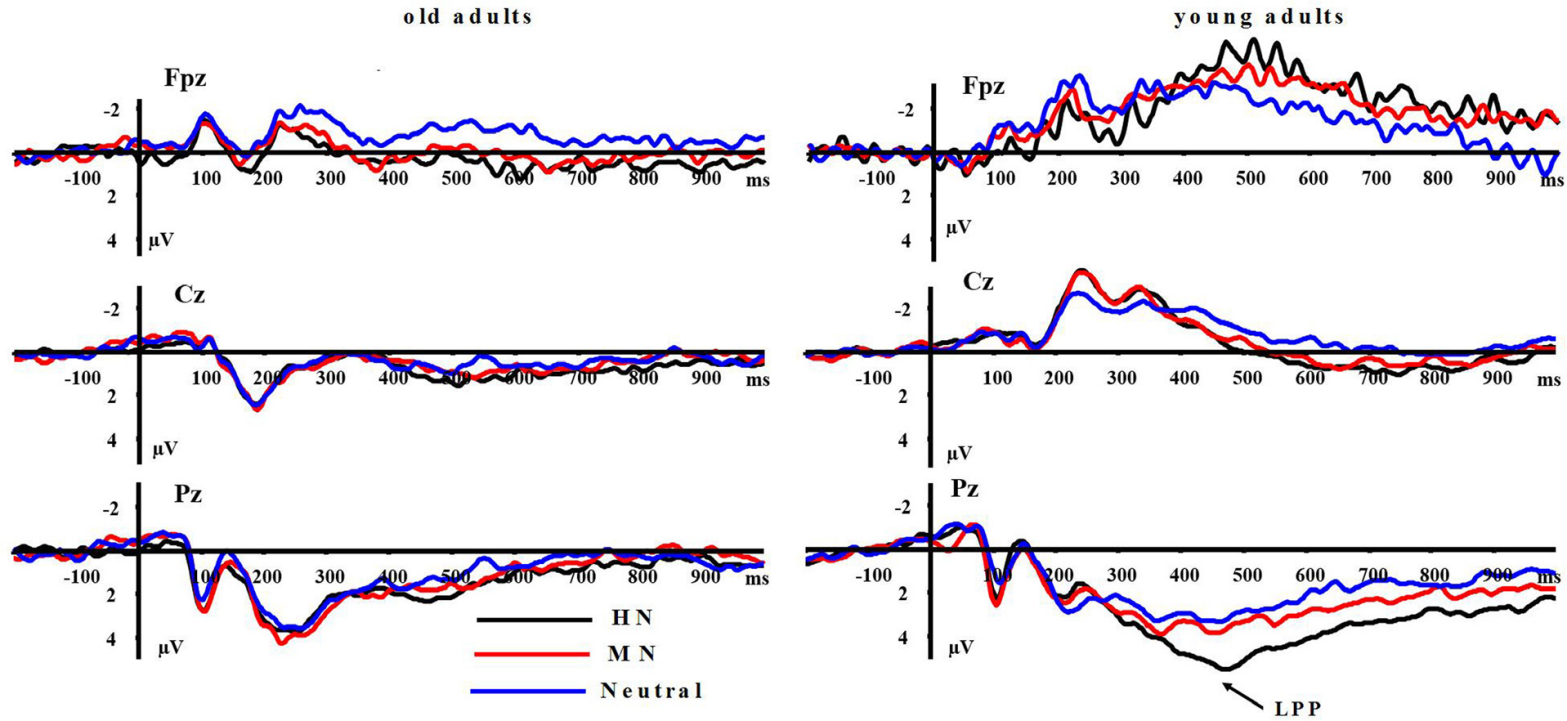
**Averaged ERPs at FPz, Cz, and Pz sites for highly positive (HP), mildly positive (MP), and neutral stimuli in young and old adults**.

**FIGURE 5:**
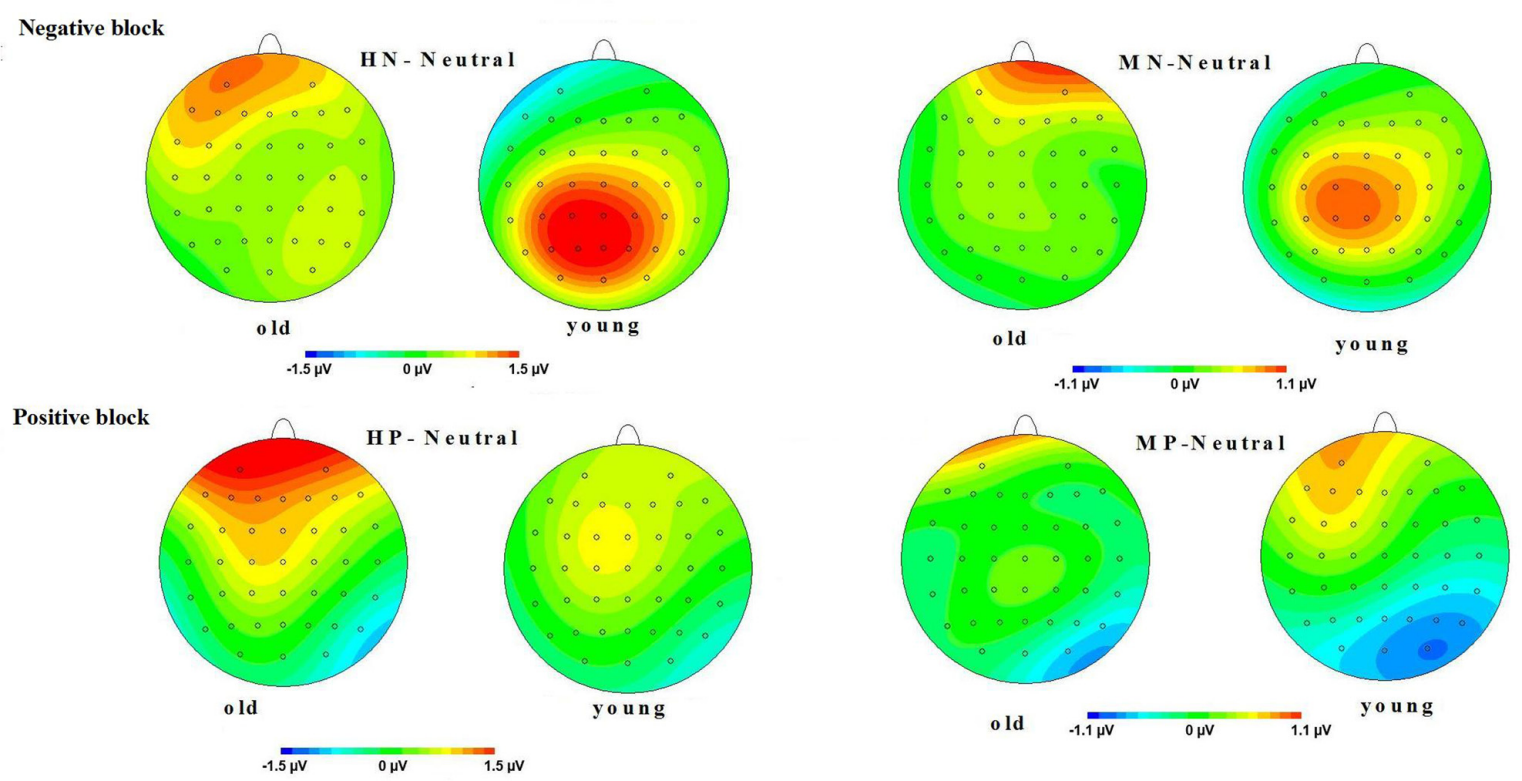
**(Top)** Topographical maps of the mean amplitudes difference (across 500–800 ms) between highly negative (HN) and neutral stimuli, and between mildly (MN) and neutral stimuli in old and young adults. **(Bottom)** Topographical maps of the mean amplitudes difference (across 500–800 ms) between HP and neutral stimuli, and between MP and neutral stimuli in old and young adults.

**LPP(400–1000 ms)**: A repeated measure ANOVA of the LPP amplitudes was conducted with the following factors: aging, emotion intensity, frontality, and laterality in the negative block. We observed significant main effects of frontality [*F*(2,64) = 48.513, *p* < 0.01] and emotion intensity [*F*(2,64) = 3.983, *p* < 0.05], and a significant three-way interaction amongst frontality, emotion intensity, and aging [*F*(4,128) = 6.269, *p* < 0.01]. The breakdown of the three-way interaction showed that the interaction effect between emotion intensity and aging is significant at parietal sites [*F*(2,64) = 12.11, *p* < 0.01], but not at prefrontal sites [*F*(2,64) = 0.948, *p* > 0.30] and central sites [*F*(2,64) = 0.892, *p* > 0.40]. The analysis of interaction effect between emotion intensity and aging at parietal sites showed a significant main effect of emotion intensity in young adults [*F*(2,32) = 16.042, *p* < 0.001]. HN stimuli elicited larger amplitudes than MN stimuli [*F*(1,16) = 9.374, *p* < 0.01] which, in turn, elicited larger amplitudes than Neutral stimuli [*F*(1,16) = 9.617, *p* < 0.01]. In contrast to young adults, old adults showed no significant amplitude differences across the three conditions [*F*(2,32) = 1.295, *p* > 0.20]. Consistent with our prediction, these results suggest that the impact of aging on the brain processing of negative stimuli, as reflected in LPP, occurs at parietal scalp sites (see **Figures 4** and **5**)

**FIGURE 6:**
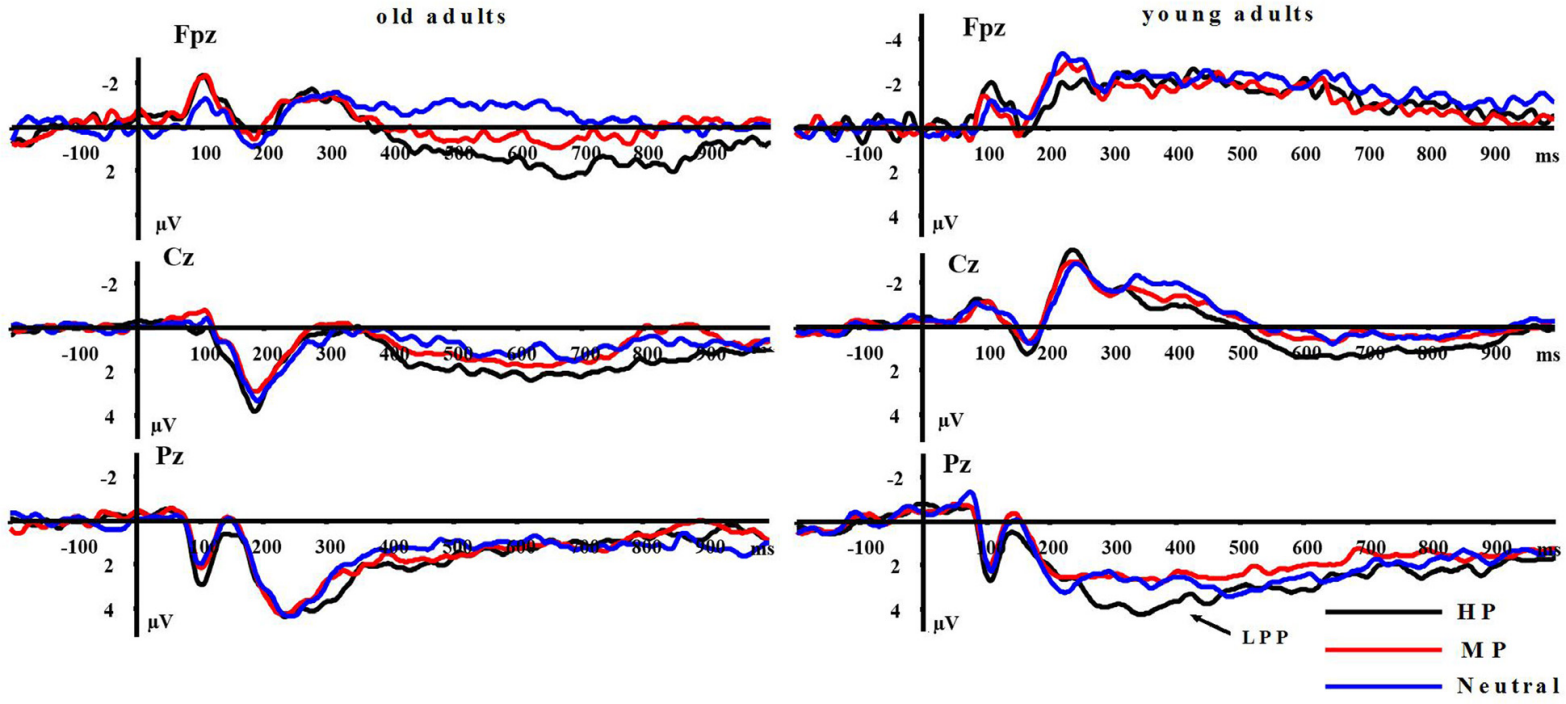
**Averaged ERPs at FPz, Cz, and Pz sites for HP, MP, and neutral stimuli in young and old adults**.

Moreover, the same ANOVA model was used to test the scalp distribution of the aging by emotion intensity interaction in the positive block. We observed significant main effects of frontality [*F*(2,64) = 41.808, *p* < 0.01] and emotion intensity [*F*(2,64) = 5.014, *p* < 0.05], and a significant three-way interaction amongst frontality, emotion intensity and aging [*F*(4,128) = 3.315, *p* < 0.05]. The breakdown of the three-way interaction showed a significant interaction effect between emotion intensity and aging at prefrontal sites [*F*(2,64) = 3.743, *p* < 0.05], instead of central sites [*F*(2,64) = 0.184, *p* > 0.70] and parietal sites [*F*(2,64) = 0.734, *p* > 0.40]. The analysis of interaction effect between emotion intensity and aging at prefrontal sites showed a significant main effect of emotion intensity in old adults [*F*(2,32) = 8.278, *p* < 0.05], and HP stimuli elicited larger amplitudes than MP stimuli [*F*(1,16) = 5.294, *p* < 0.05] which, in turn, elicited larger amplitudes than Neutral stimuli [*F*(1,16) = 5.736, *p* < 0.01]. In contrast with old adults, young adults showed no significant amplitude differences across three conditions [*F*(2,32) = 1.145, *p* > 0.2]. Consistent with our prediction, these results suggest that the impact of aging on the brain processing of positive stimuli, as reflected in LPP, occurs at prefrontal sites (see **Figures 5** and **6**).

In order to explore the timing features of the above aging-related emotional intensity effects for positive stimuli in LPP amplitudes, we conducted further ANOVA of LPP amplitudes at prefrontal region with the following factors: aging (two levels), emotion intensity (three levels) and timing (three levels: 400–600 ms, 600–800 ms, 800–1000 ms). The results showed no significant three-way interaction effect amongst aging, timing and emotion intensity [*F*(4,128) = 2.544, *p* > 0.05] in the positive block. In the positive block, the aging by emotion intensity interaction was similarly significant in 400–600 ms [*F*(2,64) = 4.388, *p* < 0.05], 600–800 ms [*F*(2,64) = 3.408, *p* < 0.05] and 800–1000 ms [*F*(2,64) = 4.619, *p* < 0.05].

Also, the same analysis was used for LPP amplitudes in the negative block. The results showed no significant three-way interaction amongst aging, timing and emotion intensity [*F*(4,128) = 1.121, *p* > 0.3]. Similarly, the two-way interaction between aging and emotion intensity was similarly significant in 400–600 ms [*F*(2,64) = 3.966, *p* < 0.05], 600–800 ms [*F*(2,64) = 4.384, *p* < 0.05] and 800–1000 ms [*F*(2,64) = 3.811, *p* < 0.05].

These results suggest that the aging-related emotional effects in LPP amplitudes exist reliably, unaffected by the time windows of LPP quantification.

#### Is the Aging-Related Emotion Effect Specific to Late Processing Stage?

The above results implied that the impact of aging on brain responding to positive and negative stimuli of varying emotional intensities was significant in LPP but not in P1 stage. In order to test the reliability of this timing effect, we conducted an ANOVA with timing (two levels: P1 and LPP), aging (two levels: young and old) and emotion intensity (three levels: high, mild, and neutral) as factors, in the negative block and in the positive block, respectively. The results showed a significant three-way interaction amongst timing, aging and emotion intensity in the positive [*F*(2,64) = 3.681, *p* < 0.05] block with the aging by emotion intensity interaction significant at LPP but not in P1 amplitudes. Similarly, there was a significant three-way interaction involving timing, aging and emotion intensity in the negative block [*F*(2,64) = 3.785, *p* < 0.05], with the aging by emotion intensity interaction significant in LPP but not in P1 amplitudes. These results confirmed that the impact of aging on brain processing of negative and positive stimuli was specific to late rather than early processing stage.

#### Perceived Frequency Report

Firstly, all subjects (*n* = 34) reported the perception of negative pictures in the negative block and positive pictures in the positive block, respectively.

**FIGURE 7:**
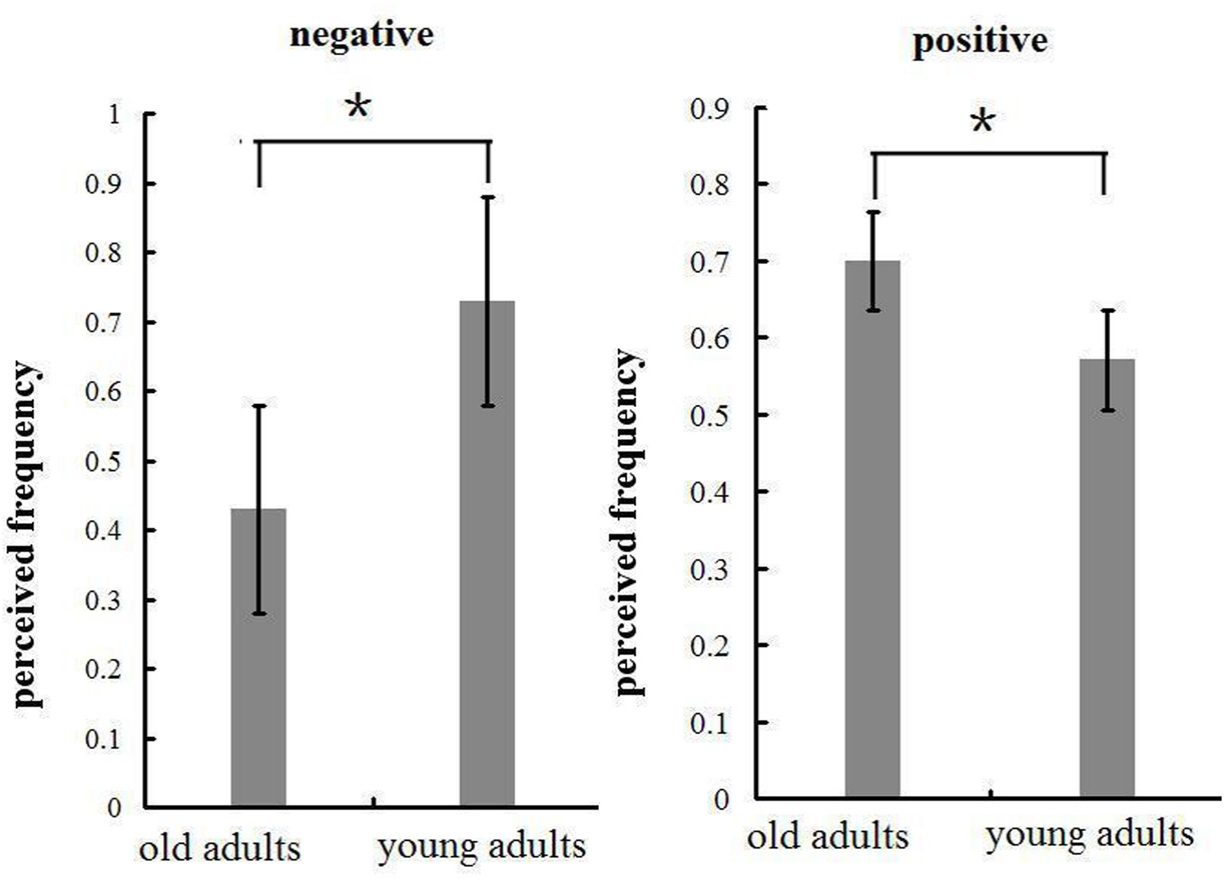
**Means and SE of perceived frequency of the emotional pictures in negative and positive blocks.** ^∗^*p* < 0.05.

**FIGURE 8:**
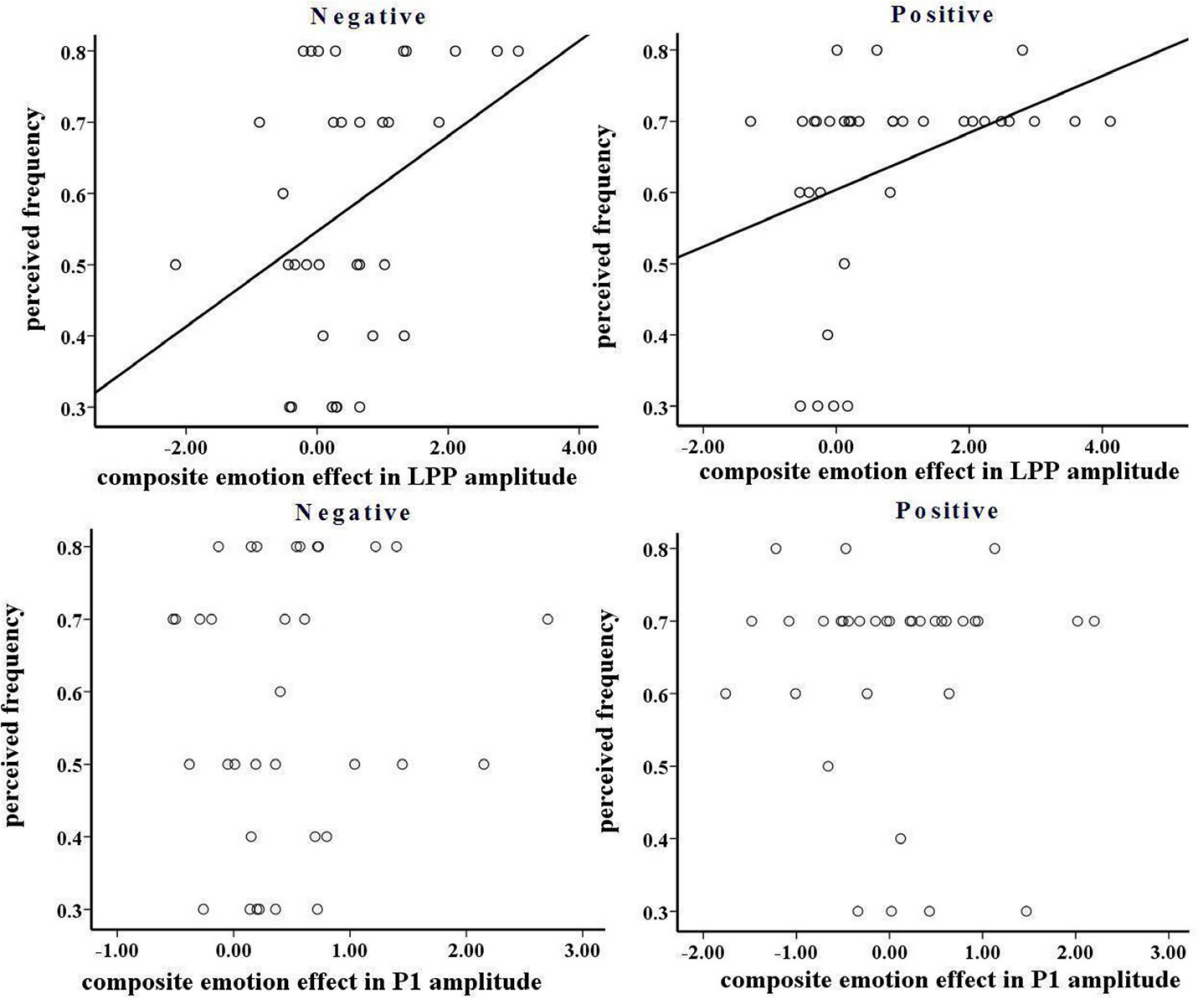
**(Top)** The correlation between composite emotion effect in late positive potentials (LPP) amplitude and perceived frequency in negative and positive blocks. **(Bottom)** The correlation between composite emotion effect in P1 amplitude and perceived frequency in negative and positive blocks.

In the negative block, the perceived frequency of negative pictures was higher in young (72.9%) relative to old adults [42.9%; *F*(1,32) = 6.571, *p* < 0.05, see **Figure 7**]. This suggests that negative stimuli influenced young adults to a greater extent compared to old adults. We included all the participants and conducted a correlation analysis between the perceived frequency and the composite emotion effect at P1 and LPP components. The composite emotion effect was defined as the average of the emotion effect for HN and MN stimuli, which was calculated as the amplitude differences between negative and neutral conditions collapsed across the corresponding electrode sites (three occipital sites for P1, and three parietal sites for LPP). The result showed a significant positive correlation between the composite emotion effect in LPP amplitudes and the perceived frequency of negative pictures (*r* = 0.368, *p* < 0.05, see **Figure 8**), whereas the correlation between the perceived frequency and the composite emotion effect in P1 amplitudes was not significant (*r* = 0.115, *p* = 0.517). This suggests that the LPP amplitude may be a unique reflection of the subjective emotion effect in the current study. Therefore, ERP data and the behavioral data both displayed decreased negative emotional effect in old adults than in young adults.

In the positive block, the perceived frequency of positive pictures was higher in old adults (70%) compared to young adults [57.1%; *F*(1,32) = 67.161, *p* < 0.01; see **Figure 7**]. This suggests that old adults may have experienced more pleasant feelings for positive pictures than young adults. In addition, we conducted a correlation analysis between the perceived frequency and the composite emotion effect in LPP amplitudes. The composite emotion effect was defined as the average of the emotion effect for HP and MP stimuli, which was calculated as the amplitude differences between positive and neutral conditions collapsed across the corresponding electrode sites (three occipital sites for P1, and three prefrontal sites for LPP). The analysis showed a significantly positive correlation between the composite emotion effect in LPP amplitudes and the perceived frequency data (*r* = 0.371, *p* < 0.05, see **Figure 8**), whereas the correlation between the emotion effect in P1 amplitudes and the perceived frequency was statistically non-significant (*r* = –0.038, *p* = 0.832), suggesting that the LPP amplitude is probably a unique reflection of the subjective emotional effect for positive stimuli. Therefore, both ERP data and the behavioral data displayed increased positive effect in old adults than in young adults.

## Discussion

P1 component is an early component peaking about 100 ms post stimulus, and it has been accepted to reflect exogenous and automatic sensory processing (Scott et al., 2009; Dan and Raz, 2012). P1 amplitudes are thought to be sensitive to attention allocation (Luck et al., 1994; Smith et al., 2003; Brown et al., 2010), and be heightened for emotional stimuli compared to neutral stimuli (Scott et al., 2009; Dan and Raz, 2012). We observed a significant emotion intensity effect but not significant emotion intensity by aging interaction for P1 amplitudes, in both positive and negative blocks. Both young and old adults showed larger amplitudes for HN and MN stimuli relative to neutral stimuli. This suggests that negative stimuli elicited an enhanced allocation of early sensory attention in both samples, and this early visual encoding of negative stimuli was similar for both groups. This result is in line with a couple of studies reporting no aging effect in early encoding of threatening information (Hahn et al., 2006; Mather and Knight, 2006), and is supported by the evidences that the aging-related positive effect results from the controlled instead of the automatic processing stage (Mather and Knight, 2006; Leclerc and Kensinger, 2008b). On the other hand, HP stimuli elicited larger P1 amplitudes compared to Neutral stimuli in both samples, and there was also no significant emotion intensity × aging interaction in P1 amplitudes for the positive block. This suggests that HP stimuli elicited enhanced early visual attention than neutral stimuli, consistent with prior findings (Holmes et al., 2009; Yuan et al., 2014a) and this enhancement was similar for young and old adults. This finding is consistent with prior behavioral studies suggesting that old adults, like young adults, showed a rapid detection of arousing information, irrespective of valence (Hahn et al., 2006; Mather and Knight, 2006; Leclerc and Kensinger, 2008b).

It is worth noting that, previous studies showed an impact of visual acuity on the processing of emotional stimuli (Butler et al., 2009). Although all the subjects reported no visual problems and found no difficulty reading textual instructions on the monitor at a distance of 2.5 m, it is still a question whether visual acuity in old adults is different from that in young adults, and whether visual acuity is an alternative interpretation of our results, as we had no quantitative measurement of visual acuity across groups. However, if visual acuity in old adults differs from that in young adults, we should have observed a significant aging effect in P1 amplitude, which has been established to reflect early visual processing of stimuli (Scott et al., 2009; Dan and Raz, 2012). However, the present study observed neither main effect of aging, nor aging by emotion intensity interaction, suggesting that the visual acuity was most likely not significantly different across old and young adults.

Distinct from P1 analysis, the analysis of LPP amplitudes showed a significant block, emotion intensity, and aging interaction. LPP amplitudes increased with negative intensity in young adults, but not in old adults. LPP activity is considered to reflect consciously controlled processing of stimulus meanings (Wood and Kisley, 2006; Kisley et al., 2007). The LPP amplitudes have been shown to increase with the allocation of voluntary attention to emotional stimuli (Hajcak and Dennis, 2009; Langeslag and Van Strien, 2010). This association is confirmed by our findings of a significant correlation between the emotion effect in LPP amplitudes and the perceived frequency of negative pictures. Young adults displayed prominent emotion effects for HN stimuli and, of a smaller size, for MN stimuli, while old adults showed no emotion effect for these stimuli. Though this result is supported by our behavioral findings that young adults reported more perception of negative stimuli than old adults, this result appears inconsistent with previous studies that old adults exhibited larger LPP amplitudes for negative compared to neutral stimuli in emotional assessment tasks (Wood and Kisley, 2006; Kisley et al., 2007). It is worth noting that the current study used a non-emotional distracting task, which might have facilitated old adults disengaging attention from negative stimuli, thus leading to reduced brain reactions to these stimuli (Isaacowitz et al., 2006a,b; Mauss et al., 2007). This hypothesis needs to be directly explored in future studies by testing the impact of aging on the attentional disengagement from negative stimuli.

However, the above findings are consistent with the following abundant evidences. It has been reported that old adults are better than young adults in sustaining positive emotions and terminating negative emotions (Carstensen et al., 2000). For example, Carstensen et al. (2000) observed that old adults had greater ability of differentiating between distinct categories of emotions than did young adults, which was thought to be linked with less neuroticism and better emotion control. This is consistent with later studies suggesting that aging is associated with habitual attention shifting from negative to positive cues (Carstensen and Mikels, 2005; Isaacowitz et al., 2006a). One explanation for these phenomena is that in their age advancement process old adults might have learned more about dealing with negative events, consequently they could spend less time on negative events when compared with young adults (Scheibe and Carstensen, 2010).

On the other hand, this study observed increasing LPP amplitudes with the pleasant intensity of positive stimuli in old adults, where young adults showed no significant emotional effects for HP and MP stimuli. This aging-related difference was observed in prefrontal but not central and parietal regions. Leclerc and Kensinger (2008a) observed greater neural activations of old adults, but not young adults, for positive compared to negative pictures in ventromedial prefrontal cortex during the analysis of picture meanings. Consistent with this finding, Ritchey et al. (2011) observed enhanced ventrolateral and medial prefrontal cortex activity in response to positive versus negative stimuli in old adults, during elaborative processing of picture meanings. Also, recent studies have shown that the function of Anterior Cingulated Cortex (ACC) is well-maintained in old adults (Fjell et al., 2009), and the engagement of rostral ACC in voluntary attention for happy faces is correlated with the old adults’ emotional stability (Brassen et al., 2011). These evidences suggest that prefrontal cortical regions play a critical role in old adults’ enhanced cognitive processing of pleasant stimulus meanings. As stated above, LPP represents elaborative, cognitive processing of stimulus meanings with voluntary attention to emotional stimuli (Kisley et al., 2007; Hajcak and Dennis, 2009; Langeslag and Van Strien, 2010). This probably explains why old adults exhibited enhanced LPP amplitudes for positive relative to neutral stimuli in prefrontal but not other scalp regions. In addition, the old adults’ enhanced LPP amplitude for positive stimuli probably reflects increased positive emotion induction, as evidenced by the higher frequency report of positive stimuli in old versus young adults, and by the positive correlation between LPP and the perceived frequency of positive pictures. Also, this argument is supported by the positive correlation between LPP amplitudes and emotion experience in many prior studies (Hajcak and Nieuwenhuis, 2006; Flaisch et al., 2008; MacNamara et al., 2011; Yuan et al., 2014a). These behavioral and electrophysiological data consistently suggest that the old adults may have experienced more positive emotions than young adults, though the two samples viewed the same pictures.

The current study used a distracting task which required subjects to count the number of pictures, irrespective of emotionality. This means that emotional processing was unlikely to have happened with full involvement of cognitive resources; but evidently, it may have occurred in the service of conscious perception and controlled processing resources. Under such a task setting, the current study observed aging by emotion interactions in LPP but not in P1 amplitudes. These results suggest that, observing an aging-related positivity effect may not request full cognitive resources, but instead just requires the involvement of conscious awareness. This view is in line with many studies reporting that old adults show an attentional preference for positive stimuli and attention disengagement from negative stimuli, once the emotional nature of the stimulus has been discerned (Isaacowitz et al., 2006b; Allard and Isaacowitz, 2008; Leclerc and Kensinger, 2008b; Allard et al., 2010).

The aging-related differences in LPP amplitudes for emotional stimuli may be explained by SST (Carstensen, 1992; Carstensen et al., 2000). This theory posits that young adults perceive their time remaining in life to be expansive and are motivated to acquire knowledge, whereas old adults perceive their time left in life as limited thus prioritize present-oriented emotional meanings. This motivational shift leads old adults to focus more on positive aspect of life. However, it has yet to be determined whether changes in time perspective do, as proposed by SST, serve as a mechanism mediating the enhanced attention of old adults to positive information. This issue needs to be explored in future studies by testing the mediation of time perspective in the association between aging and the LPP responding to emotional stimuli.

## Conclusion

By using ERP technique and a block-design covert emotional task, the present study investigated neural mechanisms underlying the aging-related enhancement of positive affects. Although the same number of emotional pictures was presented for young and old adults in each block, old adults reported more perception of positive stimuli and less perception of negative stimuli than young adults. ERP results showed larger LPP amplitudes for HN and MN stimuli compared to neutral stimuli in young adults, but not in old adults. By contrast, old adults displayed significant emotion effects for HP and MP stimuli in LPP amplitudes, both of which were absent in young adults. These results were supported by the post-experiment stimulus assessment, which showed more positive ratings of Neutral and MP stimuli, and reduced arousal ratings of HN stimuli in old compared to young adults. These behavioral and electrophysiological data consistently suggest that aging is linked with enhanced attention bias for positive stimuli and reduced susceptibility to negative stimuli, which might contribute to the enhanced positive affects and LS (see the Supplementary Material) in old relative to young adults.

## Conflict of Interest Statement

The authors declare that the research was conducted in the absence of any commercial or financial relationships that could be construed as a potential conflict of interest.

## Appendix A. Identification Numbers of CAPS Pictures Presented in this Study

HN:142,146,147,148,149,150,152,173,179,193,196,205,218,222, 232,240,243,246,248,254,255,256,268,269,276,280,281,282,283, 284,443,458,471,484,502,511,522,528,532,533,536,539,540,541, 542,555,559,569,571,572,573,577,580,583,590,597.

MN:153,155,157,161,166,169,171,184,186,195,197,198,200, 204,213,223,227,228,241,242,247,259,260,263,274,363,456,462, 466,480,507,512,519,520,524,526,531,546,549,550,552,563,567, 581,588,591,592,595,599,607,609,612,613,615,621,625.

Neutral(negative):295,297,298,304,307,311,312,313,315,316, 319,320,322,324,329,347,367,372,386,387,389,393,396,398,403, 407,411,419,422,425,426,436,455,465,490,649,658,673,674,682, 705,706,709,723,725,726,733,735,742,744,745,747,768,808,826, 834.

MP:1,2,21,460,848,5,34,38,41,50,58,60,61,63,71,87,111,119, 123,137,291,294,299,300,339,357,377,438,446,454,501,635,643, 645,757,764,765,766,787,788,789,802,810,850,8,19,24,32,33,47, 54,68,82,309,680,689.

HP:28,4,7,10,11,12,13,14,15,16,18,20,43,52,53,57,88,94,98,99, 101,102,109,118,121,129,463,478,486,488,491,663,675,691,701, 718,749,750,752,756,762,775,776,780,781,791,44,45,461,487, 640,641,642,800,813,822.

Neutral(positive):235,258,292,293,295,298,302,305,833,310, 311,318,320,327,329,336,355,381,382,386,387,403,406,410,424, 436,451,464,479,503,665,747,767,768,778,831,849,851,459,469, 504,510,514,829,517,523,534,603,601,719,732,763,746,785,842, 839.
