# Supplementary material for: The neural mechanisms underlying the aging-related enhancement of positive affects: electrophysiological evidences
Source: Front Aging Neurosci. 2015 Aug 6;7:143. doi: 10.3389/fnagi.2015.00143 (PMC4527238; doi:10.3389/fnagi.2015.00143)

In addition, we conducted a correlation analysis between the subjects' life satisfaction and the composite emotion effect at P1 and LPP components in both negative and positive blocks. In the negative block, the result showed a significant negative correlation between the composite emotion effect in LPP amplitudes and the subjects' life satisfaction (r=-0.35, p<0.05, see Figure.9), whereas the correlation between the perceived frequency and the composite emotion effect in P1 amplitudes was not significant (r=-0.019, p=0.915). In the positive block, the result showed a significant positive correlation between the composite emotion effect in LPP amplitudes and the subjects' life satisfaction (r=0.403, p<0.05, see Figure.9), whereas the correlation between the perceived frequency and the composite emotion effect in P1 amplitudes was not significant (r=-0.026, p=0.882).

We computed the correlation between the positive effect in stimulus frequency report and the subjects' life satisfaction. The positive effect in perceived stimulus frequency was defined as the differences between the perceived frequency of positive pictures in the positive block and that of negative pictures in the negative block. The result showed a significant positive correlation between the positive effect in perceived frequency and life satisfaction(r=0.227, p<0.05, see Figure.9). Therefore, the above results consistently suggest that the higher life satisfaction in old adults may result from enhanced attention bias for positive stimuli and reduced susceptibility to negative stimuli relative to young adults.


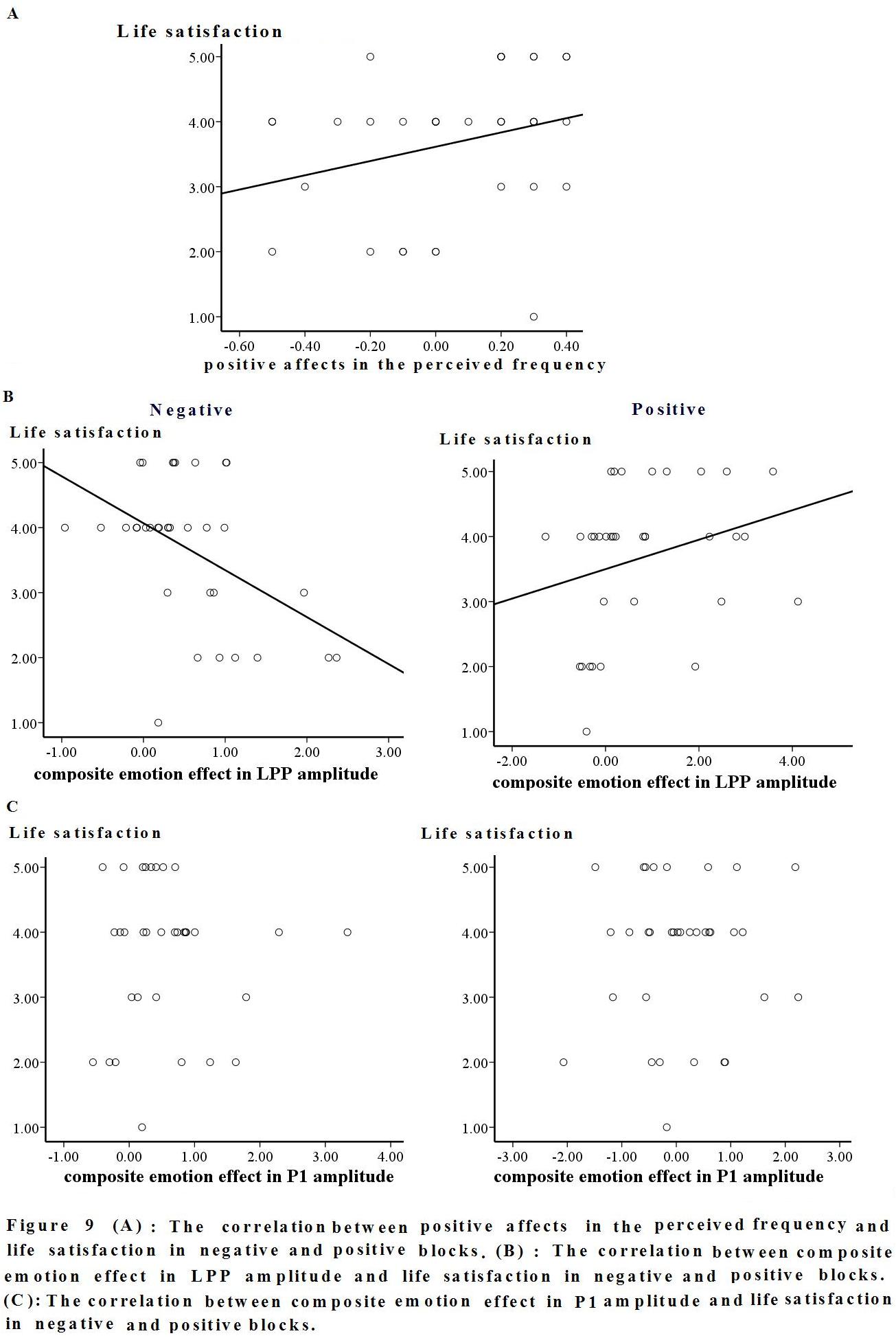

Supplement: Supplementary file 1 [file Data_Sheet_1.DOC]
